# Evolution of Genome-Organizing Long Non-coding RNAs in Metazoans

**DOI:** 10.3389/fgene.2020.589697

**Published:** 2020-11-30

**Authors:** América Ramírez-Colmenero, Katarzyna Oktaba, Selene L. Fernandez-Valverde

**Affiliations:** ^1^ Unidad de Genómica Avanzada (Langebio), Centro de Investigación y de Estudios Avanzados del IPN, Irapuato, México; ^2^ Unidad Irapuato, Centro de Investigación y de Estudios Avanzados del IPN, Irapuato, México

**Keywords:** evolution, conservation, long-non-coding RNAs, chromatin conformation, three-dimensional chromatin conformation, genome topology, gene expression regulation

## Abstract

Long non-coding RNAs (lncRNAs) have important regulatory functions across eukarya. It is now clear that many of these functions are related to gene expression regulation through their capacity to recruit epigenetic modifiers and establish chromatin interactions. Several lncRNAs have been recently shown to participate in modulating chromatin within the spatial organization of the genome in the three-dimensional space of the nucleus. The identification of lncRNA candidates is challenging, as it is their functional characterization. Conservation signatures of lncRNAs are different from those of protein-coding genes, making identifying lncRNAs under selection a difficult task, and the homology between lncRNAs may not be readily apparent. Here, we review the evidence for these higher-order genome organization functions of lncRNAs in animals and the evolutionary signatures they display.

## Introduction

The three-dimensional (3D) organization of DNA in the cell nucleus has become a significant subject of study, particularly its influence on gene regulation. Recent advances in chromatin conformation capture (3C) techniques, computational, and modeling approaches have made its study feasible on a genome-wide scale, giving insight into the structure and the dynamics of chromatin folding in space and time. Nuclear 3D organization has multiple levels and varies between cell types and biological conditions. For instance, chromosomes are subdivided into topologically associating domains (TADs) within which chromatin loops bring together regulatory elements and target loci separated in the linear genome (Dixon et al., 2012). These chromatin interactions are crucial for precise gene expression regulation (reviewed in Furlong and Levine, 2018; Schoenfelder and Fraser, 2019; Ibrahim and Mundlos, 2020). Importantly, changes in transcriptional programs result in variation in chromatin interactions within TADs, while TAD boundaries delimiting these domains are preserved (Dixon et al., 2015). TADs segregate in the nuclear space into transcriptionally active (A) and inactive (B) compartments. A/B compartments correlate well with histone modifications characteristic of euchromatin and heterochromatin, respectively, and are described as cell type-specific, being able to undergo switches during cell differentiation and lineage commitment (Lieberman-Aiden et al., 2009; Rao et al., 2014; Dixon et al., 2015; Fortin and Hansen, 2015).

In addition to DNA and histones, RNA is a major component of the cell nucleus (Rinn and Chang, 2012). High-throughput sequencing methods have revealed the pervasive transcription of thousands of non-coding RNA (ncRNA) molecules in the genome. Among the latter, long non-coding RNAs (lncRNAs) have emerged as important gene regulators in eukaryotes. lncRNAs are broadly defined as transcripts longer than 200 nucleotides, with little to no protein-coding potential (Mercer et al., 2009; Wang and Chang, 2011; Derrien et al., 2012). lncRNAs are more lowly expressed (Hezroni et al., 2015), display more tissue-restricted expression patterns (Necsulea et al., 2014), have fewer exons, and are shorter than protein-coding genes (Hezroni et al., 2015). In animals, several lncRNAs are essential to phenomena such as gene silencing, activation, and chromatin remodeling, with significant roles in development, immunity, and cancer (Guttman et al., 2011; Schmitt and Chang, 2016; Delás et al., 2017). lncRNA functions may predate the origin of metazoans, as several unicellular holozans possess lncRNAs that are distinct in terms of their histone marks as well as expression throughout their life cycle (Gaiti et al., 2017).

## Signatures of Conservation in LNCRNAs

There has been a long debate on whether most lncRNAs are functional or not (van Bakel et al., 2010; Clark et al., 2011; Lindsay et al., 2013). This discussion was, in part, sparked by the fact that the sequence of lncRNAs is generally poorly conserved across species, suggesting that they are not under purifying selection (Babak et al., 2005; Ponjavic et al., 2007; Marques and Ponting, 2009). There are several examples of orthologous RNAs that preserve their function, but whose sequence is so divergent, they can no longer be identified as orthologs by sequence similarity alone (Ponjavic et al., 2007; Ulitsky et al., 2011; Ulitsky, 2016). Thus, the detection of conservation beyond sequence is paramount to annotate candidate lncRNAs for further functional characterization.

The conservation signals in lncRNAs can differ from those typically found in protein-coding genes (Diederichs, 2014; Ulitsky, 2016). For instance, conventional conservation analyses applied to coding sequences, such as calculating the rate between synonymous and non-synonymous mutations, are not suitable for these elements. Nevertheless, lncRNAs display some sequence conservation, generally in short sequence islands, potentially due to selection constraints on sequences necessary for interacting with other transcripts, proteins, or DNA (Kapusta and Feschotte, 2014; Quinn et al., 2016; Ulitsky, 2016). lncRNAs may also display constraints on the post-transcriptional processing of the transcript, leading to the conservation of splice sites across different species (Nitsche et al., 2015; Ulitsky, 2016). lncRNAs can also possess structural conservation – a constraint that may not be readily detectable at the sequence level (Smith et al., 2013; Tavares et al., 2019). Finally, lncRNAs can have positional conservation, and be expressed from syntenic loci despite having lost most or all sequence conservation. These modes of conservation are not mutually exclusive and may be present in a single lncRNA.

Beyond their apparent lack of conservation, many functionally characterized lncRNAs modulate the organization of higher-order chromatin structures in the nucleus (Saxena and Carninci, 2011; Marchese and Huarte, 2014). lncRNAs are involved in the formation of DNA loops and domains (Wang and Chang, 2011; Zhang et al., 2014), interchromosomal structures (Hacisuleyman et al., 2016), heterochromatic regions (Deng et al., 2009; Engreitz et al., 2013), subnuclear bodies (Mao et al., 2011), and the dynamic assembly of protein complexes (Tsai et al., 2010; Lin et al., 2014; Marín-Béjar et al., 2017). Several novel experimental methods allow the identification of lncRNAs binding to chromatin *in vivo* across the genome (Li et al., 2017; Sridhar et al., 2017; Bell et al., 2018; Bonetti et al., 2020; Gavrilov et al., 2020). Recruiting and binding to effector molecules is a prevalent mode of action of lncRNAs in both *cis* and *trans* activities.

Here, we summarize lncRNAs that affect, establish, or maintain three-dimensional chromatin organization in metazoans and the conservation signals that indicate they are under selection.

## LNCRNAs That Affect Tad Conformation and Their Conservation

### Sequence Conservation

**Figure 1:**
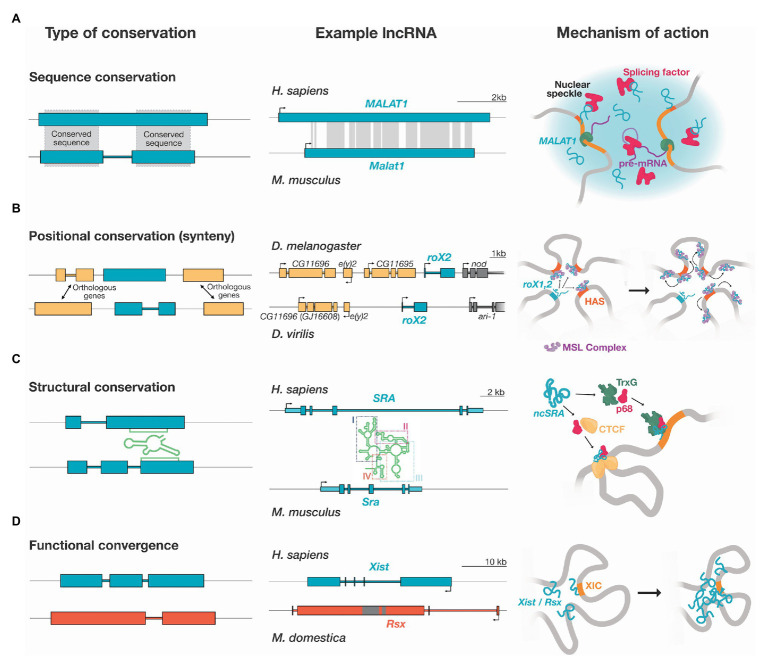
Types of conservation and mechanism of action of example lncRNAs. Diagrams show exons (big filled boxes) and introns (colored links) of lncRNAs genes. 5' and 3' UTRs are shown as light blue boxes in **(C)**. **(A)** Sequence conservation: Some lncRNAs present high levels of sequence conservation (gray shading). For example, the *Metastasis Associated in Lung Adenocarcinoma Transcript 1 (MALAT1)* lncRNA is highly conserved from human to zebrafish. Regions of conservation are shown according to the “Vertebrate Multiz Alignment & Conservation” track of the UCSC genome browser. *MALAT1* localizes to nuclear speckles, nuclear bodies for co-transcriptional and post-transcriptional pre-mRNA processing. In humans, *MALAT1* regulates the phosphorylation of serine/arginine splicing factors, enriched at nuclear speckles. **(B)** Positional conservation: lncRNAs can have a conserved genomic position but very low sequence conservation. This is the case for the *roX* lncRNAs in *Drosophila*, identified by a combination of synteny, microhomology, and secondary structure. *roX1* (not shown) and *roX2* spread to high-affinity sites (HASs), landing regions of male-specific lethal (MSL) complex, in close spatial proximity, regulating local chromatin remodeling, leading to the increased expression of genes for dosage compensation. **(C)** Structural conservation: lncRNAs can fold into a conserved secondary structure. The steroid receptor RNA activator (SRA) gene produces both a protein and a lncRNA (ncSRA). A simplified representation of the structure of the human ncSRA, as determined by Novikova et al. (2012), is depicted. ncSRA consists of four main domains, three of which are well-conserved at sequence across 36 vertebrate species and contain covariant base pairs. Different segments of the structure have differences in sequence conservation, and specific helices are highly conserved. ncSRA binds to several proteins including: trithorax group (TrxG), DEAD-box RNA helicase 5 (DDX5 or p68), and CCCTC-binding factor (CTCF), potentially acting as a scaffold for the assembly of ribonucleoprotein complexes. **(D)** Functional convergence: lncRNAs with no common origin can have an equivalent function. The *X-inactive specific transcript* (*Xist*) and *RNA on the silent X* (*Rsx*) lncRNAs act on the process of dosage compensation in different species. Both *Xist* and *Rsx* are expressed form the X inactivation center (XIC) and are spread along the X chromosome to inactivate it.

Sequence conservation in lncRNAs can range from very high to almost non-existent. Despite being generally presented as poorly conserved, a subset of lncRNAs can present significant sequence conservation across species (Necsulea et al., 2014; Hezroni et al., 2015). However, sequence conservation does not guarantee functional equivalence; a highly conserved lncRNA can be fundamental in one species while dispensable in others. For example, the lncRNA *Metastasis Associated in Lung Adenocarcinoma Transcript 1* (*MALAT1*) is highly conserved from human to zebrafish (Figure 1A; Hutchinson et al., 2007; Lin et al., 2007). While the human *MALAT1* functions in nuclear speckles, regulating alternative splicing (Hutchinson et al., 2007; Tripathi et al., 2010), cell-cycle associated genes (Yang et al., 2011), and cancer progression (Gutschner et al., 2013), the murine ortholog is neither essential for these functions nor mouse development (Eißmann et al., 2012; Nakagawa et al., 2012; Zhang et al., 2012).

However, it is more common for lncRNAs to have short conserved motifs or domains that are important for their association with DNA or proteins that regulate chromatin conformation. For example, lncRNAs that affect 3D genome topology and arise from highly conserved syntenic loci, such as the *Hox* clusters, display contrasting patterns of sequence conservation compared to their protein counterparts in the same cluster. *Hox* genes, organized in mammals in four clusters (*HoxA*–*HoxD*), encode transcription factors crucial for patterning along the anterior-posterior axis. Numerous ncRNAs are transcribed from the human HOX loci, and their expression relates to differential histone marks and transcriptional accessibility (Rinn et al., 2007).

The *HOX antisense intergenic RNA* (*HOTAIR*) lncRNA is transcribed from the boundary between domains with differential chromatin marks at the *HOXC* locus but acts in *trans* repressing transcription of coding and non-coding genes on the *HOXD* locus (Rinn et al., 2007). A chromatin loop established between *HOTAIR* locus and the *HOXC* distal enhancer (HDE) located downstream of *HOTAIR* promotes transcription of the lncRNA. This loop is disrupted by the recruitment of hepatocyte nuclear factor 4-α (HNF4α), a master regulator of epithelial differentiation, to the HDE (Battistelli et al., 2019). *HOTAIR* exists across mammals, albeit poorly conserved in sequence; it is only highly conserved in primates (He et al., 2011). Noteworthy, a highly conserved domain in exon 6, possibly the backbone of *HOTAIR*, appeared first in kangaroos suggesting the *ab initio* generation of *HOTAIR* in marsupials (He et al., 2011). Despite its low sequence conservation across mammals, key secondary structural elements of HOTAIR contain protein-binding motifs and have significant conservation or covariation (He et al., 2011; Somarowthu et al., 2015). However, studies evaluating the functional conservation of murine *HOTAIR* (*mHotair*) present contradictory results. On the one hand, the deletion of the *HoxC* cluster, including *mHotair*, did not affect *HoxD* silencing *in vivo* (Schorderet and Duboule, 2011). In contrast, mice homozygous for *mHotair* KO presented homeotic spine transformation and malformation of metacarpal bones, and derived fibroblasts showed altered expression and levels of epigenetic marks at hundreds of genes, including *HoxD* genes (Li et al., 2013). Interestingly, human and mouse *HOTAIR* differ in number, arrangement, and degree of sequence conservation among their exons. The absence of exons with protein-binding motifs in *mHotair* may partially explain differences in their function.

Another lncRNA expressed from HOX clusters is *HOXA transcript at the distal tip* (*HOTTIP*), transcribed from the 5' end of the HOXA locus in mammals and conserved in avians (Wang et al., 2011). Chromosomal looping brings *HOTTIP* into spatial proximity to its target genes in *cis*, allowing *HOTTIP* to activate transcription by binding the WD repeat domain 5/mixed lineage leukemia (WDR5/MLL) complex, driving H3K4me3 (Wang et al., 2011). *HOTTIP* and its association with CCCTC-binding factor (CTCF), which delineates active and inactive TADs within the *HOXA* cluster, also influence the expression of *HoxA* genes (Narendra et al., 2015; Wang et al., 2018).

Long non-coding RNAs also enable the establishment of inter-chromosomal structures. The *Functional intergenic repeating RNA element* (*Firre*) is a lncRNA involved in pluripotency, hematopoiesis, and adipogenesis (Hacisuleyman et al., 2014; Lewandowski et al., 2019). *Firre* accumulates across a ~5 Mb domain around its transcription site on the X chromosome (Hacisuleyman et al., 2014), located between two TADs, and highly enriched in CTCF binding sites, required for *Firre* transcription (Barutcu et al., 2018). This domain colocalizes with five regions on different chromosomes that contain genes with roles in adipogenesis. The formation of this structure depends on the interaction of *Firre* with Heterogeneous Nuclear Ribonucleoprotein U (HNRNPU), through a 156-bp repeating RNA domain (RRD; Hacisuleyman et al., 2014). This RRD is unique to *Firre*, and functions as a lineage-specific nuclear retention signal in mice and humans. The RRD and other local repeats (LRs) are conserved to different extents across *Firre* orthologs in mammals. *Firre* is also required for the super-loop formation of the inactive X chromosome (Xi), H3K27me3 deposition, and the localization of the Xi to the perinuclear region (Yang et al., 2015; Barutcu et al., 2018).

The 3D architecture of TADs enables a group of multi-exonic lncRNAs, termed *immune gene-priming lncRNAs (IPLs)*, to direct the active priming of the promoters of immune genes, necessary for a rapid and robust pro-inflammatory response as part of trained immunity (Fanucchi et al., 2019). Upon induction of transcription of immune genes by the tumor necrosis factor (TNF), chromatin contacts increase TNF-induced genes and the lncRNAs loci. *IPLs* are somewhat conserved between mouse and human; the majority possess an Alu element in their first intron and share putative transcription-factor binding motifs at their promoters.

The region comprising an *IPL*, *Upstream master lncRNA of the inflammatory chemokine locus* (*UMLILO*), engages in chromosomal contacts with CXCL chemokine genes belonging to the same TAD, but *UMLILO* does not have enhancer-RNA-like characteristics. In contrast to other *IPLs*, *UMLILO* is not conserved in mice and only partially conserved in pigs, suggesting that *IPLs* are not essential across species, but have a complementary role in ensuring robust gene expression. *UMLILO* has short conserved sequence motifs and interacts with WDR5 through its conserved exon 3, directing WDR5/MLL1 to chemokine gene promoters, mediating H3K4me3. Transcription of chemokines in *UMLILO* knockdown cells was restored by insertion of another WDR5-binding lncRNA, *HOTTIP*, under the control of the *UMLILO* promoter (Fanucchi et al., 2019). The ability of *HOTTIP* to rescue the loss of *UMLILO* is an example of convergent functional evolution, as they share minimal sequence similarity.

Another group of chromatin-modifying lncRNAs arises from the syntenic *estrogen receptor 1* (*ESR1*) locus. ESR1 is strongly upregulated in cancerous cells undergoing estrogen deprivation. A cluster of ncRNAs, *ESR1 locus enhancing and activating non-coding RNAs* (*Eleanors*), are transcribed from introns in a large chromatin cluster within a TAD that contains the *ESR1* locus (Tomita et al., 2015). These *Eleanors* form a chromatin-associated RNA cloud that delineates the TAD and *cis*-activate transcription. This TAD interacts with another active TAD that contains the apoptotic transcription factor *forkhead Box O3* (*FOXO3*; Abdalla et al., 2019). Knockdown of a promoter-associated *Eleanor*, *pa-Eleanor(S)*, induced repression of the rest of the *Eleanors* and the genes within the TAD, including ESR1 (Abdalla et al., 2019). The abundant and highly conserved *Eleanor2* increases chromatin accessibility in the *ESR1* upstream region by destabilizing nucleosomes, activating *ESR1*, and is required for the formation of the RNA cloud (Fujita et al., 2020).

### Positional Conservation

Long non-coding RNAs may be expressed from syntenic loci, suggesting a common origin, but may have lost the majority of sequence conservation (Figure 1B). The functions of these lncRNAs are thought to rely primarily on their transcription (Diederichs, 2014; Ulitsky, 2016). Thus, the evolutionary signature would be expected to reside outside the transcribed region (Ulitsky, 2016). Indeed, many lncRNAs have a very conserved promoter but little to no conservation in their transcribed region (Guttman et al., 2009). A substantial difficulty in this classification is defining when sequence conservation is entirely lost. As outlined above, several lncRNAs only retain small patches of conservation considered negligible by some authors and meaningful by others.

Examples of this conundrum are dosage compensation lncRNAs in *Drosophila melanogaster* (Figure 1B). Detailed syntenic analysis of Drosophilid genomes revealed 47 new orthologs, where only 19 had been identified by sequence similarity (Quinn et al., 2016). Importantly, it was shown that the *roX* RNA itself, only its transcription, is necessary for dosage compensation (Quinn et al., 2016). Furthermore, a distant *roX* RNA ortholog rescues the loss of *roX* between two distant species (*D. melanogaster* and *Drosophila busckii*) despite almost no sequence conservation outside an eight nucleotide-long conserved patch of microhomology (Quinn et al., 2016).

A more traditional example of positional conservation is the lncRNA *antisense to Igf2r RNA non-coding* (*Airn*), required for paternal-specific silencing of imprinted genes in the *insulin-like growth factor 2* (*Igf2r*) cluster (Sleutels et al., 2002). The function of *Airn* is conserved between human and mouse despite them sharing little conserved sequence (Yotova et al., 2008). The *Igf2r* silencing function of *Airn* was shown to be dependent on transcriptional overlap and not on the transcribed RNAs themselves (Latos et al., 2012). However, recent evidence shows that this is only the case for nearby imprinted genes, as the murine *Airn* lncRNA itself is necessary for the recruitment of chromatin-modifying complexes to distant non-overlapping genes in the cluster (Andergassen et al., 2019).

### Structural Conservation

Structural conservation is potentially the most telling signal of conservation in lncRNAs, yet the most difficult to identify. The basic premise is that structural domains may be preserved despite changes in the sequence, as long as complementary base pairs are maintained.

The non-coding isoform of the steroid receptor RNA activator (SRA), *ncSRA*, has a four-domain secondary structure with varying levels of sequence conservation (Figure 1C). *ncSRA* functions as a coactivator of several human hormone receptors by modifying chromatin structure (Novikova et al., 2012). *ncSRA* associates with CTCF and the *DEAD-BOX helicase 5* (DDX5), and this association is necessary for the insulator activity of CTCF *in vivo* (Yao et al., 2010). The functional RNA structure is conserved in all mammals, while its sequence is not. Furthermore, several of the varying positions in other species show changes predicted to help stabilize its structural elements (Novikova et al., 2012).

Dosage compensation lncRNAs (see next section) show patches of structural conservation of biological importance. The Repeat A (RepA) region of *X-inactive specific transcript* (*Xist*), essential to the establishment of X chromosome inactivation, interacts with proteins such as the polycomb repressive complex 2 (PRC2; Zhao et al., 2008), ATRX chromatin remodeler (Sarma et al., 2014), and SHARP repressor protein (McHugh et al., 2015). RepA was experimentally shown to have a complex structure that is preserved despite rapid changes across mammalian evolution, strongly suggesting that this structure is indispensable for *Xist* function (Liu et al., 2017). lncRNAs involved in dosage compensation in drosophilids, *roX1* and *roX2*, have conserved boxes that correspond precisely with stems that are necessary for binding to the male-specific lethal (MSL) proteins. Domains outside these interaction zones are not conserved and lack structure (Ilik et al., 2013; Quinn et al., 2016).

*HOTAIR* has also been shown to have a complex secondary structure, with some evidence of conservation in mammals acquired from computational methods (Somarowthu et al., 2015). However, there is some debate as to whether there is enough evidence to suggest that *HOTAIR*’s structure is conserved in mammals (Rivas et al., 2017). Similarly, secondary-structure predictions on *Firre* indicated that the RRD is a highly structured domain (Nakagawa and Hirano, 2014), consistent with LRs representing potential binding platforms for the specific targeting of proteins to specific genomic regions by lncRNAs.

### Functional Convergence: The Case of Dosage Compensation lncRNAs

The lncRNAs involved in the process of dosage compensation are extraordinary examples of *de novo* emergence of novel lncRNAs of unrelated evolutionary origins (Figure 1D). A prominent example is the *Xist* lncRNA, required for dosage compensation in the sex-chromosomes of eutherians (Penny et al., 1996). Random X-chromosome inactivation in females is necessary to balance the transcriptional output to that of males. *Xist* localizes at the X inactivation center (XIC) and is expressed exclusively from the inactivated X (Xi; Brown et al., 1991). During the onset of X inactivation, *Xist* accumulates at the XIC (Clemson et al., 1996), and then targets gene-rich regions that are spatially close to its transcription site (Engreitz et al., 2013; Simon et al., 2013), incorporating them into the *Xist* silencing domain and spreading further to cover the complete future Xi (Engreitz et al., 2013). *Xist*-mediated inactivation involves the transcriptional silencing of most genes on the Xi, and its compaction and recruitment to the nuclear lamina (Zhao et al., 2008; Hasegawa et al., 2010; Chu et al., 2015; McHugh et al., 2015; Minajigi et al., 2015).

While exonic sequences of *Xist* are well-conserved among eutherians, there are differences in the exon-intron structure, length, and sequence between species (Nesterova et al., 2001; Elisaphenko et al., 2008). This indicates that either *Xist* genes present a high adaptation level or that their sequence and structure are not essential (Elisaphenko et al., 2008). *Xist* is not present in non-eutherian vertebrates, including marsupials, despite common epigenetic features on the Xi, such as loss of active histone marks and exclusion of RNA polymerase II (Chaumeil et al., 2011). Homology of *Xist* with promoters and exonic sequences of the protein-coding gene ligand of numb-protein x 3 (*Lnx3*) found in marsupials, chicken, and fish suggests that *Xist* emerged through pseudogenization of *Lnx3*, possibly by the insertion of tandem repeats from transposable elements (Duret et al., 2006; Elisaphenko et al., 2008).

Interestingly, in marsupials, X-chromosome inactivation is imprinted, tissue-specific, and somewhat incomplete compared to eutherians, and thought to be achieved by female-specific expression of the lncRNA *RNA on the silent X* (*Rsx*), which is transcribed from and coats the paternal chromosome (Grant et al., 2012). The independent evolution of *Xist* and *Rsx* adds to the notion of dosage systems rapidly evolving from ancient silencing mechanisms common to all eukaryotes through the use of lncRNAs (Gendrel and Heard, 2014; Graves, 2016). The discoveries on the regulation of *Xist* by non-coding elements located at its own and the neighboring TAD and the impact of this 3D conformation on the regulatory landscape adds another layer of complexity to the mechanisms for dosage compensation (van Bemmel et al., 2019; Galupa et al., 2020).

lncRNAs are also the effectors of dosage compensation in drosophilids, but they differ in both origin and mechanism to those in mammals. Here, the *roX1* and *roX2* lncRNAs mediate the upregulation of genes on the single male X chromosome to equalize expression of the two X chromosomes in females. *roX1* and *roX2* associate to the MSL proteins, forming the MSL complex that localizes to numerous specific sites along the male X (Franke and Baker, 1999), mediating histone acetylation and increasing transcription. The MSL complex does not alter the global architecture of the X chromosome, but it does spread *via* spatial proximity from high-affinity sites – enriched at TAD boundaries – to other regions (Ramírez et al., 2015). Contrary to *Xist*, whose activity is limited to the chromosome from which it is expressed (Wutz and Jaenisch, 2000), *roX* transgenes target the X chromosome *in trans* and rescue *roX1* and *roX2* mutant males (Meller and Rattner, 2002).

The independent origin of *Xist* in mammals, *Rsx* in marsupials, and *roX1* and *roX2* in flies suggests that lncRNAs may be one of the fastest mechanisms to evolve novel epigenetic controls. As these lncRNAs participate in dosage compensation but have emerged independently in several lineages, they are extraordinarily difficult to identify as functionally convergent. Additional examples of functionally equivalent lncRNAs with no evolutionary relationship may likely have gone undetected.

## Discussion

Distinctly, lncRNAs have emerged as an additional layer of complexity involved in shaping the three-dimensional organization of the genome by interacting and modifying the structure of chromatin. Several lncRNAs affect chromatin conformation and display a combination of conservation signals that may be difficult to identify solely by looking at traditional genomic conservation metrics (summarized in Table 1). These signatures could prove useful to identify and prioritize lncRNA candidates for experimental functional characterization. Sequence conservation can be identified using traditional computational sequence comparison methods. Recent examples have shown that conserved sequence stretches can be much shorter in lncRNAs than in protein-coding sequences, highlighting the need to look for tiny stretches of sequence conservation (microhomology; Quinn et al., 2016). Positional conservation of lncRNAs can be identified using multiple genome alignments complemented with transcriptomic data that support the existence of non-coding transcripts in multiple taxa. The detection of splice site conservation uses a similar approach but focuses on identifying splice sites *via* modeling or direct RNA-seq evidence, followed by comparison across taxa (Nitsche et al., 2015). In the case of structural conservation, covariation signatures in multiple sequence alignments may indicate the conservation of a structure (Nawrocki et al., 2009; Gruber et al., 2010; Will et al., 2012). One of the most significant limitations is the difficult problem of distinguishing covariation from sequence conservation. Thus, these methods can better identify conserved structures in highly varying sequences in diverse and multiple taxa (Rivas et al., 2017, 2020).

**Table 1:** Characterized long non-coding RNAs (lncRNAs) that are involved in nuclear genome topology.

lncRNA	Function	Mode of action	Interacting proteins	Association with chromatin topology	Conservation	References
*Xist*	X chromosome inactivation in mammals	*In cis*	PRC1, PRC2, HNRNPU, RBM15, SHARP, WTAP, HNRNPK, LBR, and many others	The organization of the XIC into two topologically associating domains (TADs) ensures the proper interaction of *Xist* and its antisense lncRNA *Tsix* with regulatory elements	Present only in eutherian mammals. Presence of common core exonic sequences, despite species-specific unique sequences, and variation in length and gene structure	Nesterova et al., 2001; Plath et al., 2003; Elisaphenko et al., 2008; Zhao et al., 2008; Hasegawa et al., 2010; Engreitz et al., 2013; Chu et al., 2015; McHugh et al., 2015; Minajigi et al., 2015; Moindrot et al., 2015; Chen et al., 2016; Pintacuda et al., 2017; van Bemmel et al., 2019; Galupa et al., 2020
				During X inactivation, *Xist* spreads along the chromosome exploiting the three-dimensional (3D) organization, resulting in compaction and recruitment to the nuclear lamina		
*HOTTIP*	Gene control of HOXA locus for distal identity	*In cis*	WDR5/MLL and CTCF	A chromatin loop gets *HOTTIP* into spatial proximity to HOXA genes. Associates with CTCF to define functional TADs at HOXA cluster	Portions conserved in mammals and avians	Wang et al., 2011, 2018
*Airn*	Its transcription prevents overexpression of Igfr2 locus in a paternal-specific matter	*In cis*	EHMT2	Forms an RNA cloud, creating a repressive domain	Tandem direct repeats at the CpG island at 5' end are conserved in human and mouse at an organizational level but not by sequence	Lyle et al., 2000; Seidl et al., 2006; Nagano et al., 2008; Latos et al., 2009, 2012; Koerner et al., 2012; Santoro et al., 2013
*Kcnq1ot1*	Silencing at imprinted Kcnq1 locus in a paternal-specific manner	*In cis*	EHMT2, PRC2, PRC1, and DNMT1	Formation of repressive chromatin loop on the imprinting control region of the locus	Well-conserved motifs between human and mouse	Pandey et al., 2004, 2008; Mancini-Dinardo et al., 2006; Mohammad et al., 2008, 2010; Zhang et al., 2014
*pRNA*	Mediates silencing by CpG methylation of rRNA genes at nucleolus *via* DNA:RNA triplex formation	*In cis*	NoRC and DNMT3b	Establishment of nucleolar heterochromatin	Conserved across eutharians, various levels of sequence conservation, and highly conserved secondary-structure motifs	Mayer et al., 2006, 2008; Santoro et al., 2010; Schmitz et al., 2010;Guetg et al., 2012; Jacob et al., 2013; Savić et al., 2014; Wehner et al., 2014
*LUNAR1*	IGF1 signaling, promotes cell proliferation in cancers	*In cis*	None reported	A chromatin loop that brings into contact the promoter of *LUNAR1* and the enhancer of IFG1R is necessary for the expression of both genes, which reside in the same TAD	Not reported	Trimarchi et al., 2014; Peng and Feng, 2016
*Khps1*	Activates transcription of its sense proto-oncogene SPHK1, *via* DNA:RNA triplex formation at SPHK1 enhancer	*In cis*	EP300	Khps1 transcription leads to a transcriptionally active open chromatin state by recruitment of EP300/CBP, transcripton of KHPS1 enhancer and eviction of CTCF	Conservation between humans and rodents	Imamura et al., 2004; Postepska-Igielska et al., 2015; Blank-Giwojna et al., 2019
*UMLILO*	Trained immune response on chemokine genes	*In cis*	WDR5/MLL	In chemokine TAD, chromosomal looping brings the super-enhancer region harboring UMLILO into contact with chemokine genes, allowing UMLILO RNA to guide WDR/MLL to the promoters to facilitate H3K4me3 epigenetic priming	Partial conservation between human, chimpanzee, and pig, absent in mouse	Fanucchi et al., 2019
*Eleanors*	Activation of ESR1 locus, apoptosis resistance	*In cis*	None reported	*Eleanors* RNA cloud delineate ESR1 TAD and activate transcription	Varying levels of conservation for each *Eleanor*	Tomita et al., 2015; Abdalla et al., 2019; Fujita et al., 2020
*HOTAIR*	Represses expression in HOXD locus and other genes, including imprinted	*In trans*	PRC2, RCOR1, and AR	*HOTAIR* transcripts demarcate silent and active domains in HOXD locus.	Poorly conserved by sequence, secondary structure motifs conserved between mouse and human	Rinn et al., 2007; Gupta et al., 2010; Tsai et al., 2010; Li et al., 2013; Somarowthu et al., 2015; Zhang et al., 2015; Portoso et al., 2017
*ncSRA*	Activation of steroid receptors (isoforms of SRA code for protein)	*In trans*	SRC-1, PRC2, TrxG, NANOG, CTCF, SHARP, DDX5, and others	Diverse, chromatin looping and modification as scaffold for proteins in both active and inactive domains	Significant sequence conservation and high structural conservation	Lanz et al., 1999; Shi et al., 2001; Zhao et al., 2007; Yao et al., 2010; Novikova et al., 2012; Wongtrakoongate et al., 2015
*roX1* and *roX2*	Dosage compensation in *Drosophila*	*In trans*	MSL Proteins	The MSL complex (*rox* + MSL proteins) has high affinity sites on TAD borders	There are roX orthologs across drosophilids	Franke and Baker, 1999; Park et al., 2008; Ilik et al., 2013; Maenner et al., 2013; Ramírez et al., 2015; Quinn et al., 2016
*IPW*	Repression of maternally expressed genes. Possibly implicated in Prader-Willi syndrome	*In trans*	EHMT2	Allele-specific formation of heterochromatin at DLK1–DIO3 region.	Poorly conserved by sequence between human and mouse	Wevrick et al., 1994; Wevrick and Francke, 1997; Stelzer et al., 2014
*Firre*	Role in adipogenesis, nuclear architecture, inflammatory response (*in vitro*), and hematopoiesis (*in vivo*)	*In trans*, but occupies domain *in cis*	HNRNPU	*Firre* acts as a scaffold for the formation of an inter-chromosomal structure. Locates at border of TAD in a CTCF binding region. Required for super loop formation of inactive X	Conservation across mammals, high convergence of repeating domain in primates. Local Repeats are conserved between species of the same order	Hacisuleyman et al., 2014, 2016; Yang et al., 2015; Lu et al., 2017; Barutcu et al., 2018; Lewandowski et al., 2019
*TERRA*	Implicated in telomeric and subtelomeric heterochromatin formation, stability and maintenance	*In cis* (to telomeres) and *in trans*	Shelterin components (TERF1 and TERF2), ORC1, CBX5 NoRC, ATRX, POT1, and others	*TERRA* transcription depends on chromosome looping	Telomere transcription is conserved across vertebrates and *Saccharomyces cerevisiae*	Azzalin et al., 2007; Luke et al., 2008; Schoeftner and Blasco, 2008; Deng et al., 2009; Postepska-Igielska et al., 2013; Beishline et al., 2017

In the context of studying novel lncRNAs, its unique conservation signatures, albeit more difficult to detect, are excellent ways to identify potentially functional lncRNA candidates and give a first insight on their possible mechanisms of action. They can also help guide the search for homologous mechanisms in other species. Complementing *in silico* studies with experimental approaches in the context of spatiotemporal gene expression programs is crucial to further assess the impact of these ncRNAs on modulating genome architecture, including their specific contribution to the complexity and evolution of animal gene regulation.

## Author Contributions

All authors participated in writing and reviewing the manuscript and approved the final version for publication.

### Conflict of Interest

The authors declare that the research was conducted in the absence of any commercial or financial relationships that could be construed as a potential conflict of interest.
